# Effects of high-intensity interval training on depressive symptoms in Hong Kong community-dwelling older adults with mild to moderate depressive symptoms: a study protocol for a cluster randomized controlled trial

**DOI:** 10.3389/fpubh.2026.1697763

**Published:** 2026-01-27

**Authors:** Yanping Wang, Wei Liang, Yanping Duan, Wui Man Benson Lau, Ngai Man Jackie Chan, Shuyan Yang, Julien Steven Baker

**Affiliations:** 1 Department of Sports and Health Sciences, Hong Kong Baptist University, Hong Kong, Hong Kong SAR, China; 2 Mindfulness Movement and Health Joint Lab, Shenzhen University, Shenzhen, China; 3 School of Physical Education, Shenzhen University, Shenzhen, China; 4 Department of Rehabilitation Sciences, Hong Kong Polytechnic University, Hong Kong, Hong Kong SAR, China; 5 Department of Innovative Social Work, City University of Macau, Macao, Macao SAR, China

**Keywords:** Baduanjin Qigong, community dwelling, depressive symproms, high-intensity interval training, older adults

## Abstract

**Background:**

Older adults with mild to moderate depressive symptoms are at high risk of developing severe depression along with complications, disability, and mortality. Early intervention during mild to moderate stages of depressive symptoms is important to prevent further deterioration. Exercise as a readily available approach has been recognised for reducing depressive symptoms. High-intensity interval training (HIIT), a novel form of exercise that has the potential to improve both depressive symptoms and physical health, has garnered increasing attention. However, the application of HIIT to address depressive symptoms among older adults remains scarce.

**Aims:**

This proposed study aims to investigate the effects of HIIT on depressive symptoms, biomarkers of depressive symptoms, physical fitness, sleep quality, and quality of life in Hong Kong community-dwelling older adults with mild to moderate depressive symptoms.

**Methods:**

This study will be a single-blinded, cluster-randomised, controlled trial comprising three groups and three assessments. The three groups will consist of a HIIT group, a Baduanjin Qigong control group, and a recreation workshop non-exercise control group. The 144 eligible participants from 9 community centres (clusters) will be randomly allocated in a 1:1:1 ratio to three groups for 16 weeks through cluster randomisation. The primary outcome measure will be self-reported depressive symptoms. The secondary outcome measures will include salivary cortisol (a biomarker of depressive symptoms), physical fitness, sleep quality, and quality of life. The outcomes will be assessed at baseline, after completion of the intervention, and at a three-month follow-up.

**Discussion:**

This study will provide valuable evidence on whether older adults in the HIIT group could gain more improvement in depressive symptoms and other health-related benefits compared to the control groups. If HIIT demonstrates superiority, it could be prescribed as a new exercise regimen to benefit older adults with depressive symptoms in future studies. The research findings may have considerable impacts on the future prevention and treatment of mental disorders and may also contribute to promoting healthy aging among Hong Kong older adults.

**Clinical trial registration:**

ClinicalTrials.gov, identifier NCT06014294.

## 1 Introduction

Depressive symptoms are a common mental health problem and contribute to morbidity and mortality in ageing populations (1–3). Previous studies have indicated a high prevalence of depressive symptoms in older adults globally (31.7%) (4). The values are greater in the Hong Kong population (32.3%) (5). Depressed older adults present with a series of common symptoms, including depressed mood, insomnia, vague somatic pains, and cognitive decline (6). Older adults with depressive symptoms often experience chronic illnesses, reduced fitness, poor sleep quality, and a decline in quality of life (7–9). Furthermore, these individuals frequently exhibit abnormal hormone patterns, particularly concerning cortisol, which may negatively impact their cognitive function and physical health (10). Those experiencing clinical depression or non-clinical depressive symptoms have been found to have a blunted cortisol awakening response or overall lower cortisol levels (11, 12). Cortisol has been proposed as a potential biomarker of both major depressive disorder and depressive symptoms in the context (12, 13). Depressive symptoms can be categorised as mild, moderate, and severe (14). Compared to people with mild to moderate depressive symptoms, those with severe conditions may develop psychotic symptoms (e.g., delusions) and have higher mortality, disability, and treatment costs (15). Effective interventions for mild to moderate depressive symptoms can prevent the deterioration of depressive pathology to severe and psychotic symptoms. Therefore, addressing this issue while considering personal, social, and economic factors for public health is particularly urgent.

Exercise has been extensively recommended as an alternative way to improve depressive symptoms among older adults (16). In addition to providing antidepressant effects similar to other approaches (pharmacological interventions, psychotherapy) (17, 18), exercise can offer substantial concurrent benefits, such as ameliorating chronic disease, increasing physical fitness, improving sleep quality, and improving quality of life (19–22). Further, exercise is cost-effective and can be offered to larger populations as it has fewer contraindications. In previous research, mind–body exercise (e.g., Baduanjin Qigong, Tai Chi, Yoga) has emerged as a compelling recommendation for improving depressive symptoms among older adults when compared with aerobic exercise, strength training, multi-component exercise, and exergaming (23–25). Four meta-reviews have indicated that mind–body exercise typically involves low to moderate intensity and longer durations, emphasising slow movements, breath regulation, and achieving mind–body balance (23–26). However, the long duration (≥ 60 min/session in most mind–body exercises) (26), slow pace, and cognitive demand may discourage depressed older adults from maintaining the interventions (26–28). Although studies have recommended low-intensity mind–body exercise for health benefits with high adherence among older adults, evidence has shown that high-intensity exercise can yield more improvement in depressive symptoms and physical function after exercise intervention, especially for older adults with depression (29–31).

High-intensity interval training (HIIT) is an increasingly popular exercise modality that includes repeated bouts of high-intensity exercise, typically lasting seconds to minutes, interspersed with rest periods (32). Recent research has demonstrated the potential antidepressant effects of HIIT interventions by modulating brain-derived neurotrophins, dopamine, and the homeostasis of the HPA axis, through enhanced cardiovascular and cerebrovascular function. This suggests a biological mechanism for HIIT interventions in the prevention and treatment of depressive symptoms (33). Psychologically, accomplishing short intensive workouts can alleviate depressive symptoms by helping individuals divert from rumination and fostering a sense of mastery, self-efficacy, and resilience (34–37). Moreover, HIIT appears to be a promising approach showing greater effects on cardiorespiratory function and physical fitness than low to moderate-intensity exercise (38). In addition, a scoping review of HIIT programs among older adults found that HIIT was generally safe, well-tolerated, and associated with high adherence (31).

Over the past 20 years, most HIIT research has focused on adolescents (39, 40), adults (32, 41), healthy older adults and older adults with heart disease (31, 42). No previous HIIT study has investigated older adults with mild to moderate depressive symptoms. In addition, some studies have demonstrated methodological weaknesses, including inadequate control groups for exercise, small sample sizes (from 11 to 38 participants in the HIIT group) (41, 43, 44). Additionally, most HIIT programs have been conducted in a laboratory environment, and depressive symptoms were only examined as a secondary outcome. These research findings may limit the evaluation and application of HIIT interventions on depressive symptoms when generalising to a real-world setting. Therefore, there is an urgent need to investigate the efficacy of HIIT on depressive symptoms and whether it can yield superior effects compared to mind–body exercise and a non-exercising control group among older adults in community settings.

The current study aims to evaluate the effectiveness of a HIIT intervention program on depressive symptoms among Hong Kong older adults with mild to moderate depressive symptoms compared with Baduanjin Qigong and a non-exercise control. The following Hypotheses will be tested in this study: (1) participants in the exercise groups (HIIT group; Baduanjin Qigong group) would have greater improvements in self-reported depressive symptoms, a biomarker of depressive symptoms (salivary cortisol), physical fitness, sleep quality, and quality of life than the non-exercise control group (recreation workshop group) at post-intervention and 3 months after the intervention; (2) participants in the HIIT group would have greater improvements in self-reported depressive symptoms, biomarker of depressive symptoms (salivary cortisol), physical fitness, sleep quality, and quality of life than the Baduanjin Qigong control group at post-intervention and 3 months after intervention.

## 2 Methods

The study protocol was approved by the Ethics Committee of Hong Kong Baptist University (approval number: REC/21–22/0169). The trial will comply with the Helsinki Declaration and the international Good Clinical Practice Guidelines (45). A written informed consent form will be collected from each participant. Personal information about potential and enrolled participants will be protected confidentially.

### 2.1 Study design

**Figure 1:**
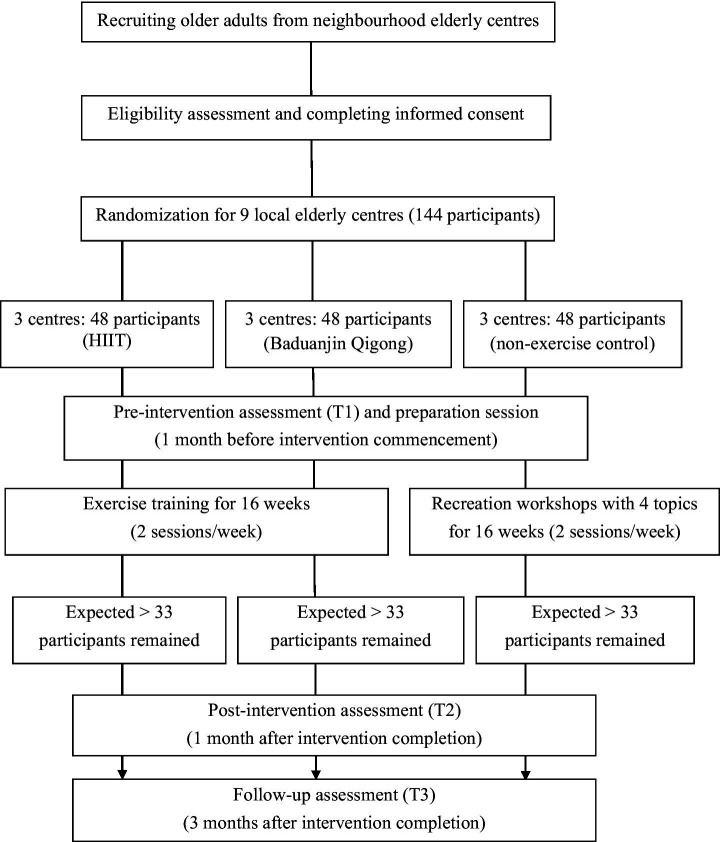
Consort flow diagram.

This study will be a single-blinded three-group cluster-RCT to examine the effects of HIIT intervention on depression symptoms, physical fitness, sleep quality, and quality of life immediately after a 16-week intervention and the residual effects 3 months following completion of the intervention. A cluster random sampling method will be adopted to recruit the participants. With elderly centres used as a cluster unit for randomization and intervention, nine elderly centres in Hong Kong will be randomly assigned to three groups. Thus, participants will be randomly recruited and assigned through elder centres to one of three groups: a HIIT group, an exercise control group (Baduanjin Qigong), and a non-exercise control group (recreation workshops). Randomisation will be conducted using the random function available in Excel by a researcher who will not be involved in the participant recruitment and data collection. The block size for randomization will be 3, and the clusters within each block will be sorted by block number and a random number. Evaluation will be conducted at pre-intervention (T1), post-intervention (T2), and 3 months after intervention completion (T3). Outcome assessors will be blinded to the intervention assignment results. The CONSORT flow diagram (46) is presented in Figure 1.

### 2.2 Study participants

The target participants will be community-dwelling older adults with mild to moderate depressive symptoms in Hong Kong. The following selection criteria will be employed to screen for eligibility: (1) 60 to 74 years at the start date of the project; (2) Chinese version of Geriatric Depression Scale (GDS-15) scoring 5–11 (i.e., mild to moderate level of depressive symptoms) (47); (3) pass the PAR-Q + screening (48) or with the physician’s approval for readiness of participation in the high-intensity exercise; (4) no severe chronic diseases, such as coronary heart disease, chronic heart failure, chronic obstructive pulmonary disease, osteoarthritis, which are diagnosed clinically in the medical history; (5) reach the average level of 2-min step test (aerobic fitness; ≥ 94 counts for male and female), 30 s sit and stand (lower limb strength; ≥ 15 counts 94 for male and female), and the timed up and go fitness test (mobility and dynamic balance; ≤ 5.9 s for male and female) showing physical competence for high-intensity exercise, based on the fitness norm of Chinese community-dwelling older adults (49); (6) no cognitive impairment, as determined by the Chinese version of the Mini-Mental Status Examination (i.e., score ≥ 24) (50); (7) No previous substantial experience in practicing HIIT or Baduanjin Qigong. A signed written informed consent form will be collected from each participant.

### 2.3 Sample size estimation

According to government data, each neighbourhood elderly centre in Hong Kong must have at least 800 members per year (Social Welfare Department, https://www.swd.gov.hk/). All these conditions enable us to recruit sufficient older adults with mild and moderate levels of depressive symptoms to the centre. Hence, for a feasible study implementation principle, a cluster size of 16 was selected in the current study. An average medium effect size (Cohen’s *f* = 0.25 converted from *η*^2^ = 0.06) based on previous meta-analysis studies with HIIT interventions on depression (51), an alpha of 0.05, a statistical power of 90%, an ICC of 0.01, and a 30% attrition rate (43) are the parameters of the estimation. Sample size was calculated by G*Power 3.1.9.7 software, using repeated measures to detect the within-between interaction between ANOVA analyses of three assessments by three groups, and equation for cluster design = [1 + (n-1)*ρ*], *n* is the average cluster size, *ρ* is the intraclass correlation coefficient, which represents the correlation between observations within the same cluster (52). A total of 144 participants in 9 clusters (3 clusters with 48 participants each) will be required for this study.

### 2.4 Intervention content

#### 2.4.1 Intervention group (High-Intensity Interval Training, HIIT)

Based on previous literature, the duration of effective HIIT programs for improving mental and physical health among older adults generally ranges from 4 to 16 weeks, with at least two sessions per week (31, 51). Considering that people with depressive symptoms may need more time to obtain benefits from exercise therapies, as well as the feasibility of program implementation in older adults, an intervention period of 16 weeks with two sessions per week (a total of 32 training sessions) will be adopted in the current intervention. Each session will comprise three sections: warm-up, main exercise, and cool-down.

According to the HIIT recommendations formulated by the American College of Sports Medicine (53), the intensity of HIIT workouts will be designed at 80–90% HRmax (Borg categorical scale 5–9) to ensure safety and maximum benefit for older adults. HRmax is estimated via an age-related prediction equation [HRmax = 208-(0.7*age)] (54). Exercise intensity will be monitored by heart rate monitors (Polar Verity Sense) as well as self-reported Rate of Perceived Exertion (RPE) using a Borg categorical scale of 0–10 (55, 56). For Week 1–2, the HIIT section will include ten intervals of 30 s of HIIT work at 80–90% HRmax (RPE 5–9), separated by nine intervals of 60 s of active recovery at 50–70% HRmax (RPE 1–3). For warm-up and cool-down sections, the intensity will be set as 50–70% HRmax (RPE 1–3). The workloads of each HIIT interval will be increased by 5 s every two weeks and finally reach 19 min 45 s in Week 15–16. The total HIIT section will progress from 14 min in Weeks 1–2 to 19 min 50 s in Weeks 15–16. Given that diverse types of exercise may contribute more to the improvement of depressive symptoms compared with only a single type of exercise (57). Each HIIT section will include 10 movements of aerobic and resistance combined exercises, where each movement will be performed as quickly as possible within one interval. There is a total of 38 different movements in the HIIT program. The HIIT program will be guided by a qualified instructor from the Physical Fitness Association of Hong Kong with the assistance of student helpers. The content for the formal HIIT program is presented in Table 1.

**Table 1:** Intervention content of HIIT.

HIIT 2 sessions/week	Warm-up	HIIT section		Cool-down
Intensity	50–70% HRmax (1–3 RPE)	HIIT work intervals: 80–90% HRmax (5–8 RPE) Recovery intervals: 60–70% HRmax (2–3 RPE)		50–70% HRmax (1–3 RPE)
Week 1–2	10-min: stretching and dynamic movement	14 min 30 s HIIT work x 10 intervals + 60 s active recovery x 9 intervals	HIIT work (10 movements) (1) lunge with fast punch; (2) squat with calf raise; (3) rope skipping; (4) standing crunch; (5) star jumping; (6) straight leg touch; (7) butt kickers; (8) sumo squat and side crunch; (9) running; (10) high knees and claps. Active recovery: Step-on	10-min: Light walking, stretching, abdominal breathing, and relaxation
Week 3–4		14 min 50 s 35 s HIIT work x 10 intervals + 60 s active recovery x 9 intervals	HIIT work (10 movements) (1) *squat with a fast punch*; (2) squat with calf raise; (3) rope jump; (4) *standing side crunch*; (5) star jumping; (6) straight leg touch; (7) butt kickers; (8) *squat to front kick*; (9) running; (10) *squat with lunge*. Active recovery: Step-on	
Week 5–6		15 min 40 s 40 s HIIT work x 10 intervals + 60 s active recovery x 9 intervals	HIIT work (10 movements) (1) squat with fast punch; (2) *squat twist*; (3) *scissor jumps*; (4) standing crunch; (5) star jumping; (6) straight leg touch; (7) *skater jump*; (8) Squat to front kick; (9) running; (10) *squat with front lunge*. Active recovery: Step-on	
Week 7–8		16 min 30 s 45 s HIIT work x 10 intervals + 60s active recovery x 9 intervals	HIIT work (10 movements) (1) *cross-punches*; (2) Squat twist; (3) Scissor jumps; (4) Squat to front kick; (5) *high knees*; (6) *high knees with touch*; (7) skater jump; (8) *side-step and prison squat*; (9) running; (10) squat with the front lunge. Active recovery: Step-on	
Week 9–10		17 min 20 s 50 s HIIT work x 10 intervals + 60 s active recovery x 9 intervals	HIIT work (10 movements) (1) cross-punches; (2) *in-out jump*; (3) *lunge jumps*; (4) squat to front kick; (5) high knees; (6) high knees with touch; (7) skater jump; (8) *lateral lunges*; (9) running; (10) *squat and uppercut*. Active recovery: Step-on	
Week 11–12		18 min 10 s 55 s HIIT work x 10 intervals + 60 s active recovery x 9 intervals	HIIT work (10 movements) (1) *lateral lunge and touchdowns*; (2) in-out jump; (3) lunge jumps; (4) squat to front kick; (5) *Heisman*; (6) high knees with touch; (7) *squat jump*; (8) fast running; (9) *low skater jumps*; (10) squat and uppercut. Active recovery: Step-on	
Week 13–14		19 min 60 s HIIT work x 10 intervals + 60 s active recovery x 9 intervals	HIIT work (10 movements) (1) *slams*; (2) side-step and prison squat; (3) squat jumps; (4) *lunge and knee drive*; (5) Heisman; (6) lateral lunges and touch down; (7) *reverse lunge*; (8) *seal jacks*; (9) fast running; (10) low skater jumps. Active recovery: Step-on	
Week 15–16		19 min 50 s 65 s HIIT work x 10 intervals + 60 s active recovery x 9 intervals	HIIT work (10 movements) (1) *lunge kick*; (2) side-step and prison squat; (3) squat with calf raise; (4) lunge and knee drive; (5) *twist jump*; (6) *slam and straight leg touch*; (7) butt kicks; (8) seal jacks; (9) low skater jumps; (10) *curtsy lunge.* Active recovery: Step-on	

HIIT, high-intensity interval training; HRmax, maximum heart rate; RPE, rate of perceived exertion.

The movement in italics indicates the new movement, which differs from the previous session.

A preparation session will be conducted at the research lab one month prior to the formal HIIT program. All participants in the HIIT group (*n* = 48) will initially have their HRmax estimated using an age-related prediction equation (54). Then, participants will be thoroughly familiarized with the 38 movements of the HIIT exercise and the entire HIIT procedure. Finally, participants will be asked to implement the HIIT program (30s HIIT work x 10 intervals + 60s active recovery x 9 intervals), where they will need to adjust their individual movement speed to reach the required HIIT intensity.

#### 2.4.2 Exercise control group (Baduanjin Qigong)

For the exercise control group, Baduanjin Qigong will be applied. This is in consideration that the acceptance, feasibility, and effectiveness of Baduanjin Qigong as a mind–body exercise with low to moderate intensity to improve older adults’ depressive symptoms has been extensively demonstrated (58, 59). The exercise routine of Baduanjin includes body postures and motion, concentrative breathing, and a meditative state of mind (60). Participants will practice a set of Baduanjin exercises repeatedly during one session. The Baduanjin Qigong exercise will be taught by a qualified Baduanjin instructor from the Hong Kong Wushu Union with the assistance of student helpers. The content for the Baduanjin Qigong exercise is presented in Table 2.

**Table 2:** Intervention content of Baduanjin.

Baduanjin 2 sessions/week	Warm-up	Baduanjin section		Cool-down
Intensity	50–70% HRmax (1–3 RPE)			50–70% HRmax (1–3 RPE)
Week 1–2	10-min: stretching and dynamic movement	14 min	Practice the following 8 movements under the guidance of the instructor: Preparation posture. (1) Prop up the sky with two improved tri-jiao; (2) Draw a bow on both sides like shooting a vulture; (3) Raise a single arm to regulate the spleen; (4) Look back to treat five strains and seven impairments; (5) Shake the head and wag to expel Heart (Xin)-fire; (6) Pull toes with both hands to reinforce the kidney; (7) Clench one’s fist and glare to increase strength; (8) Rise and fall on tiptoe seven times to treat all diseases; Ending posture.	10-min: Light walking, stretching, abdominal breathing, and relaxation
Week 3–4		14 min 50 s		
Week 5–6		15 min 40 s		
Week 7–8		16 min 30 s		
Week 9–10		17 min 20 s		
Week 11–12		18 min 10 s		
Week 13–14		19 min		
Week 15–16		19 min 50 s		

HRmax, maximum heart rate; RPE, rate of perceived exertion.

#### 2.4.3 Non-exercise control group (recreation workshops)

To control the influence of confounders (e.g., social aspects of group activities), participants in the non-exercise control group will receive a series of group-based workshops related to daily recreation. The workshop sessions will be identical to the exercise groups (e.g., frequency and duration of intervention, sub-group mode) but no formal exercise treatment. It covers four topics throughout the 16 weeks: music appreciation, gourmet food appreciation, popular tourist attractions worldwide, and painting appreciation. Each workshop will include both instructor-delivered knowledge sessions and group interaction sessions. The workshop content is presented in Table 3.

**Table 3:** Intervention content of the recreation workshop.

Workshop 2 sessions/week	Topic	Time
Week 1–2	Music appreciation	34 min
Week 3–4	Music appreciation	34 min 50 s
Week 5–6	Food appreciation	35 min 40 s
Week 7–8	Food appreciation	36 min 30 s
Week 9–10	Tourist	37 min 20s
Week 11–12	Tourist	38 min 10 s
Week 13–14	Painting and handicraft	39 min
Week 15–16	Painting and handicraft	39 min 50 s

### 2.5 Intervention strategies

#### 2.5.1 Implementation strategies

Following safe, supportive, active, and enjoyable principles (61, 62), a series of strategies will be employed during the intervention: (1) Participants in each group will be divided into two subgroups (24 participants in each subgroup). The sub-groups within the same intervention condition will differ in the timeslot for attending the sessions but share the same instructor and intervention venues; (2) exercise sessions will be performed in different small teams of maximum 4 participants in one team with similar physical fitness and maximum heart rate; (3) participants in exercise sessions will wear Polar Verity Sense heart rate monitors connected to the Polar GoFit application, which will be monitored by student helpers and displayed on a tablet screen for providing real-time feedback to participants during exercise; (4) participants will be asked to record their RPE immediately after the completion of HIIT interval or Baduanjin movement; (5) HIIT sessions will be performed with continuous music and the music tempo will be set as 120 to 140 bpm for HIIT sections, and 60–80 bpm for warm-up and cool-down sections (63). For Baduanjin Qigong, traditional Chinese music will be used during the exercise; (6) any adverse events (e.g., suffering acute illness or exercise injury) that occurred during the intervention will be tackled, referred to the emergency plan, and documented.

#### 2.5.2 Recruitment and retention strategies

As suggested by previous studies with depressive older adults (64), several practical strategies will be adopted for participant recruitment and retention, including (a) emphasizing study benefits and promoting positive attitudes and beliefs for participating in the project (e.g., expected health outcomes, free physical fitness test), (b) applying several behavioural change techniques that facilitate the retention of participation (e.g., positive persuasion, verbal encouragement, WhatsApp reminder one day before the intervention session, heart rate feedback), (c) providing financial incentives and awards for completing all measurements of outcomes (HK$100 coupon for each participant who completes the measurements at each wave (T1-T3) and a total HK$1500 lucky draw award to three participants who complete all measurements of all waves), (d) arranging exercise or workshop venues near participants’ residences.

### 2.6 Outcome evaluation

Outcome evaluations will be performed three times at pre-intervention (T1), post-intervention (T2), and 3 months after intervention completion (T3). All the data collection will be implemented at the elderly centre, except for saliva collection, which will be conducted at the participant’s home.

#### 2.6.1 Primary outcome variable

##### 2.6.1.1 Depressive symptoms

The Chinese version of the 15-item Geriatric Depression Scale (GDS-C) will be used to measure depressive symptoms (47). The GDS is the most widely used scale for detecting depression symptoms in older adults (Cronbach’s *α* = 0.81–0.83). Participants will be asked 15 items, such as “Are you satisfied with your life in the past week?.” Answers are given on a yes/no scale. The total score ranges from 0 to 15, where 0–4 = normal, 5–8 = mild depressive symptoms, 9–11 = moderate depressive symptoms, and 12–15 = severe depressive symptoms (36).

#### 2.6.2 Secondary outcome variables

##### 2.6.2.1 Salivary cortisol

Previous studies have suggested that the cortisol awakening response (CAR) can be a potential biomarker of depressive symptoms in older adults (10, 13, 65). Protocols for evaluating CAR vary across the literature (66). The decision on the appropriate number of saliva samples involves a cost/accuracy trade-off: more post-awakening samples (0, 15, 30, 45, 60 min) improve the accuracy of CAR estimation but also entail higher costs and greater participant burden (67). Minimal protocol with two samples (usually 0 and 30 min) is an acceptable method for assessing CAR dynamics, as adopted by previous research (12, 67). This approach is less burdensome for older adults to maintain compliance and is sufficient for calculating the delta (Sample 2 – Sample 1). Therefore, this study will employ a two-sample protocol. Saliva samples (2–3 mL) will be collected at awakening and 30 min after awakening, respectively, to capture the dynamic increase. Delta *Δ* of cortisol between 30 min post-awakening vs. awakening levels computed as the cortisol awakening response.

All participants will be provided with a printed instruction that adheres to guideline (66) and asked to follow the assessment instructions. Saliva samples will not be taken if participants are ill or have a fever. Participants will be instructed to refrain from eating, drinking, and brushing their teeth for at least 30 min before collecting saliva. In addition, participants will be asked to report their stress levels using a 5-point scale before saliva sample collection to decrease assessment bias (68). The collection procedure will involve subjects putting a cotton swab in the mouth for at least 4 min until the swab is soaked with sufficient saliva. Then, the cotton swab with saliva is placed back into the containers. Following the collection of samples, participants will be instructed to record the exact time of collection on the label. They should store the salivary collection tubes in the freezer zone of their home refrigerator to keep the salivary cortisol stable until transported to the research lab within 24 h after collection. All the uncentrifuged saliva samples in the lab will be stored at −20 °C until analysis. Saliva will be assayed for cortisol (μg/dL) concentrations by a registered laboratory using a high-sensitivity ELISA kit (69). The inter-assay coefficients of variation (based on low- and high-control samples) are set at 5% for cortisol (70). If saliva samples do not meet the collection requirement, for example, participants only submit a sample at one collecting time point and do not meet the required saliva volume to detect cortisol concentration, then the participants will be asked to redo the test.

##### 2.6.2.2 Physical fitness

Fitness will be assessed using the Senior Fitness Test manual (71). It is a widely used measurement for physical fitness in aging populations (53). There are seven testing items measuring all the five dimensions of fitness, including body mass index (BMI), 30 s chair stand for lower limbs’ muscle strength, 30 s arm curl for upper limb muscle strength, 2-min step test for aerobic endurance, chair sit-and-reach test for lower body flexibility, back scratch test for upper body flexibility, and 8 ft. up-and-go test for mobility and dynamic balance (71). The reliability and validity of the testing items have been previously described in the Manual (71). The current study will follow the testing procedures outlined in this manual.

##### 2.6.2.3 Sleep quality

The Chinese version of the Pittsburgh Sleep Quality Index (PSQI) will be used to measure sleep quality (72). The PSQI is a widely validated scale appropriate for use with older adults (73). The scale includes 24 items, covering seven domains: subjective sleep quality, sleep latency, sleep duration, sleep efficiency, sleep disturbance, daytime dysfunction, and use of sleep medications. Each domain score ranges from 0 (no difficulty) to 3 (severe difficulty) (Cronbach’s *α* = 0.83–0.85). A total score will be calculated, with a higher score indicating poorer quality of sleep (scoring > 5 denotes the sleep disturbance).

##### 2.6.2.4 Quality of life (QoL)

The extensively validated Hong Kong Chinese WHO Quality of Life Scale Brief Version (WHOQOL-BREF) will be used to assess older adults’ QoL (74). The scale includes 28 items, covering four dimensions: physical health, psychological health, social relationships, and environment. Answers are given on a 5-point Likert scale, ranging from 1 (very poor) to 5 (very good) (Cronbach’s *α* = 0.73–0.84). The scale yields a score for each dimension and a total score, with higher scores indicating a higher quality of life.

#### 2.6.3 Other measures

Socio-demographic information will be collected at baseline as potential confounders, including gender, age, marital status, occupation, education level, type of housing, family size, and household income. In addition, common chronic diseases (diagnosed by a doctor) will be collected at baseline.

### 2.7 Process evaluation of the intervention

A process evaluation will be conducted to assess program implementation and gather participants’ perspectives on the intervention and data collection. A comprehensive framework for designing and reporting process evaluation of cluster RCTs will be used (75). The evaluation will target intervention effects, implementation, and overall satisfaction with the program using a 10-item 5-point Likert Process Evaluation questionnaire. In addition, the Exercise Acceptability Scale (76) will be used to evaluate the feasibility and acceptability of the intervention.

### 2.8 Procedure

**Figure 2:**
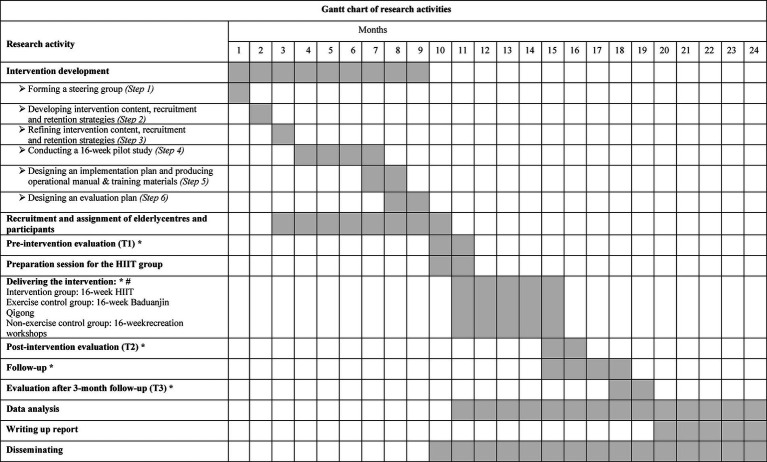
Gantt chart of research activities. *: Lengths of outcome evaluations at each time point, intervention, and follow-up will be 1 month, 4 months, and 3 months, respectively. As participant recruitment will last 2 months, an additional month has been added to each activity. #: Time schedule of intervention delivery in this figure is appropriate for participants recruited in the 10th month only. Time schedule for those recruited in month 11 will be extended accordingly.

Figure 2 demonstrates the procedure of intervention development, participant recruitment, data collection, intervention implementation, and evaluation. The proposed study will be completed over 24 months, as shown in Figure 2. In brief, (1) intervention contents and practical strategies will be developed and modified by a steering group and evaluated in a 16-week pilot study. Implementation materials and evaluation plan of the intervention will also be completed at this step; (2) all older adults from elderly centres will be invited into the study and screened for eligibility for participation; (3) all eligible participants will receive the outcome evaluations (both primary and secondary) within 1 month before the implementation of the intervention (T1). Meanwhile, the HIIT group will participate in preparation sessions during this period; (4) during the 16-week intervention, participants in three groups will receive a WhatsApp reminder one day before the intervention session. The process evaluation will be conducted for each session and the overall intervention during the intervention; (5) two more outcome evaluations will be performed within 1 month immediately after the intervention and 3 months follow-up (T2 and T3).

### 2.9 Data analysis

All data analyses will be conducted using SPSS version 29. Descriptive statistics will be used to describe the baseline characteristics of participants. The primary analyses will use intention-to-treat analysis involving all randomised participants at baseline (77). Missing data from both dropouts and completers will be imputed using a multiple imputation (MI) approach with chained equations before the analysis (78). Further, generalised linear mixed models (GLMM) will be applied to evaluate the intervention effects on outcome measures at baseline, post-intervention, and follow-up, with time, groups, and their interaction as fixed effects. The baseline values showing significant differences across groups will be controlled as covariates, while elderly clusters and individuals will be treated as random effects. *Post-hoc* tests will be conducted if a significant interaction effect is detected using the sequential Sidak method for multiple comparisons. The intra-class correlation coefficient (ICC) will be calculated to identify the variance between elderly centres. The significance level will be set as 95% (two-tailed), and the effect size Cohen’s *d* will be calculated for within and between groups, where *d* = 0.30, 0.50, 0.80, indicating a small, medium, and large effect size, respectively.

### 2.10 Pilot study

A 16-week pilot study involving all three groups will be conducted to examine and refine the intervention’s contents, strategies, and materials. Participant recruitment and maintenance, data collection instrument, adaptability, and feasibility of the intervention will also be fully considered in the pilot study. Participants will be recruited and screened from three elderly centres, each group comprising 8 eligible participants. They will receive the assigned intervention (HIIT, Baduanjin Qigong, or workshop) twice weekly for 16 weeks, along with baseline and post-intervention evaluations (including self-report questionnaires, fitness testing, and salivary cortisol). The participants in the pilot study will not be involved in the larger-scale cluster RCT study. Amendments will be made where necessary in the following larger-scale study.

## 3 Discussion

International and local data have indicated that depressive symptoms are common among older adults. Although there is increasing research addressing the effects of HIIT on depressive symptoms (41, 43, 44, 51, 79). No study has targeted community-dwelling older adults with mild to moderate depressive symptoms. To the best of our knowledge, this study, for the first time, develops and examines the effect of HIIT compared to Baduanjin Qigong and workshop on depressive symptoms in Hong Kong community older adults. It is expected that participants in the HIIT group will gain greater improvements in self-reported depressive symptoms, a biomarker of depressive symptoms (salivary cortisol), physical fitness, sleep quality, and quality of life compared to the Baduanjin Qigong control group and the non-exercise control group (recreation workshop group).

The current study findings would have important academic, practical, and policy implications. The study results would contribute to the research gap and add valuable scientific evidence to the use of HIIT research regarding the effects of HIIT on depressive symptoms in older adults with depressive symptoms. Practically, the findings of this study would provide a new and effective exercise regimen specifically for improving older adults’ depressive symptoms. It may inform the noticeable therapeutic value of HIIT and refine the physical activity recommendations for older adults with a risk of depression. Ultimately, we will propose and assist health governments in advocating and disseminating HIIT programs among all elderly centres to tackle the mental problems of older adults and further improve the mental health status among older adults at risk of depression in Hong Kong.

The study is strengthened by a three-arm cluster randomised controlled trial design. Comparison among HIIT, traditional Baduanjin Qigong, and non-exercise workshops would not only explore the effectiveness of HIIT but also examine the optimal exercise regimen for older adults with depressive symptoms. Moreover, the cluster RCT design would decrease the possibility of contamination among participants in the three groups during the intervention. Furthermore, apart from the self-reported depressive symptoms, the inclusion of a biomarker of depressive symptoms (salivary cortisol) adds a valuable dimension to this study by providing objective and biological evidence for evaluating intervention effects. In addition, the current study can examine the long-term effects of HIIT by observing a three-month follow-up, which would provide insights into the sustainability of intervention effects over time. Finally, this HIIT program conducted in dwelling-community settings (indoor activity room in the community) would answer the intervention effects in a real-world and pragmatic scenario, which indicates good external validity compared to previous HIIT interventions conducted in lab or medical settings (80, 81), making the findings more applicable to real-life situations.

To improve future research, some limitations of this protocol need to be discussed. First, the limited number of cooperative elderly centres restricts the representativeness of the cluster sample. More elderly centres covering all administrative districts in Hong Kong should be included and randomly selected (e.g., randomised by economic level and geographical location) in the future to control for enrolment bias within the cluster. Additionally, considering the age differences in the health status of older adults between the two exercise groups and the physical fitness competence requirement for high-intensity exercise, only older adults aged 60–74 will be recruited for this study. Therefore, the findings of this study may not apply to older adults aged 75 or older.

The findings will be communicated through relevant social media platforms and websites directed at pertinent practitioners and stakeholders. Academic publications will be submitted to peer-reviewed journals appropriate to their content. In addition, public presentations will be delivered at relevant national and international conferences.

## 4 Conclusion

This study will be worthwhile and valuable in developing a new and efficacious exercise regimen program for improving depressive symptoms in community-dwelling older adults. This study program is expected to be transferred to a practical antidepressant approach as a low-cost and sustainable community-based mental health service for mental health promotion among older adults in the future.
